# Extracellular Vesicle-Mediated Communication between the Glioblastoma and Its Microenvironment

**DOI:** 10.3390/cells9010096

**Published:** 2019-12-30

**Authors:** Esperanza R. Matarredona, Angel M. Pastor

**Affiliations:** Departamento de Fisiología, Facultad de Biología, Universidad de Sevilla, 41012 Seville, Spain; ampastor@us.es

**Keywords:** tumor microenvironment, exosomes, microvesicles, glioma stem cells

## Abstract

The glioblastoma is the most malignant form of brain cancer. Glioblastoma cells use multiple ways of communication with the tumor microenvironment in order to tune it for their own benefit. Among these, extracellular vesicles have emerged as a focus of study in the last few years. Extracellular vesicles contain soluble proteins, DNA, mRNA and non-coding RNAs with which they can modulate the phenotypes of recipient cells. In this review we summarize recent findings on the extracellular vesicles-mediated bilateral communication established between glioblastoma cells and their tumor microenvironment, and the impact of this dialogue for tumor progression and recurrence.

## 1. Introduction

The glioblastoma is the most aggressive primary adult brain cancer. In accordance with the World Health Organization, this tumor is classified as a grade IV glioblastoma multiforme (GBM), and is associated with very poor prognosis. Despite surgery, radiotherapy and chemotherapy, the median survival is less than 15 months [1]. Considerable research has been carried out to deepen in the understanding of the GBM biology, but still, this cancer remains incurable and lethal. 

GBMs are characterized by a strong invasive nature and a high level of cellular heterogeneity, with genetic and epigenetic differences among tumor cells of the same GBM, which makes challenging the characterization and treatment of this disease [2,3,4]. Among the tumor’s cellular heterogeneity, it contains a small population of self-renewing and highly proliferative tumor-initiating cells, called GBM stem cells (GSCs), which are resistant to treatments and are thought to be responsible for tumor progression and tumor recurrence [5,6,7]. 

GBMs recruit normal brain cells to promote their growth, invasion and nourishment in the brain. For this purpose, GBMs make use of different communication routes with the environs, which include secreted molecules, gap junctions, tunneling nanotubes and extracellular vesicles (EVs) [8]. EV-mediated communication has unique features compared to the other communication pathways mentioned, since it allows the delivery of the vesicle cargo not only in the tumor environs but also at distant sites [9,10]. In addition, the cargo includes nucleic acids that can alter gene expression in recipient cells [11]. Recent findings have demonstrated that EVs released by GBMs are able to create a tumor-supportive microenvironment through their actions on immune cells, vasculature and glial cells. In turn, cells from the tumor microenvironment also secrete EVs that can modulate GBM behavior. In this article we review recent literature showing EV-mediated bilateral communication between GBM cells and the tumor microenvironment, and we discuss its relevance for tumor progression and recurrence.

## 2. Extracellular Vesicles: Structure, Biogenesis, Secretion and Uptake

**Figure 1:**
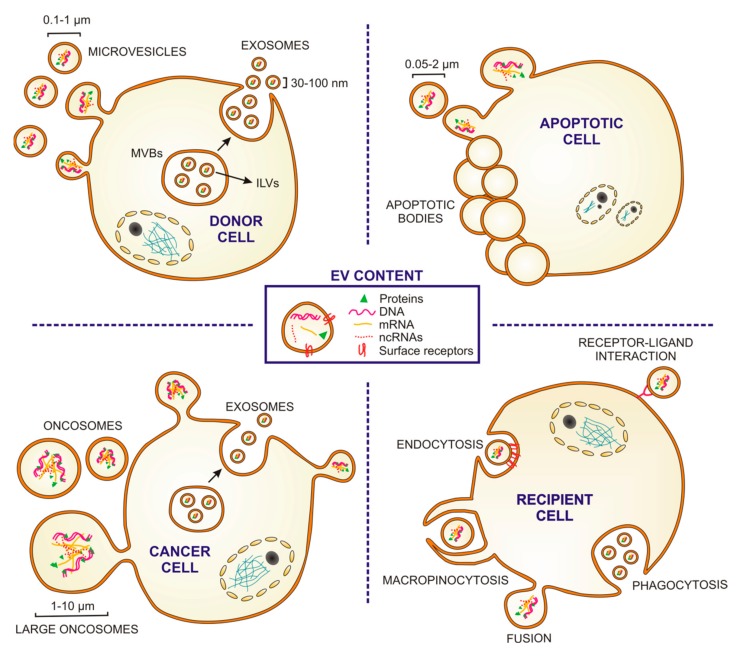
Extracellular vesicles: types, sizes, content, biogenesis and uptake. Cartoon depicting the origin and sizes of different types of extracellular vesicles (EVs). Microvesicles are released by outward budding of the plasma membrane, and exosomes from the endosomal system via fusion of multivesicular bodies (MVBs) with the cytoplasmic membrane which releases the intraluminal vesicles (ILVs) (see “DONOR CELL”). Apoptotic bodies are EVs released by membrane budding of cells dying by apoptosis (see “APOPTOTIC CELL”). Some tumor cells release oncosomes and/or large oncosomes (see “CANCER CELL”). EVs can be taken up through different mechanisms: macropinocytosis, phagocytosis, endocytosis or fusion. They can also interact with receptors in the recipient cell membrane (see RECIPIENT CELL). EVs contain cytosolic components (soluble proteins, DNA, mRNA and non-coding RNAs (ncRNAs)) and express surface molecules of their cell of origins.

EVs are phospholipid-bilayer-enclosed extracellular spherical structures, with a size range between 30 nm and 10 µm, which contain a wide variety of proteins, fragments of double-stranded DNA, mRNAs and non-coding RNAs, such as microRNAs (miRNAs), transfer RNAs and long non-coding RNAs [11,12,13]. EVs are secreted by multiple cell types and have been involved in intercellular communication between neighboring or distant cells through the transfer of genetic, protein or lipid-based messages from the host cell to recipient ones [10,11]. While in some cases, EV release is constitutive by nature, in others it is influenced by pathological situations, such as cancer, immune responses or cardiovascular diseases [14,15,16]. EVs can be formed at the plasma membrane by direct budding towards the extracellular space [17]. The plasma membrane-derived EVs have been classically called “microvesicles” (100–1000 nm in diameter) (Figure 1); they are released by many cell types during biological events of considerable diversity although the nature, and significance of the underlying process is still not completely understood [18,19]. In addition to “microvesicles,” there are additional plasma membrane-derived EVs, such as apoptotic bodies (50–2000 nm), oncosomes (100–400 nm) and large oncosomes (1–10 µm) (Figure 1) [18,20,21,22]. Apoptotic bodies are released from dying cells by blebbing and fragmentation of cell membranes in a controlled manner. Oncosomes and large oncosomes are secreted by cancer cells and contain abnormal and transforming macromolecules, such as oncogenic proteins than can promote malignant transformation in recipient cells [23,24]. Alternatively, EVs can form by an intracellular endocytic trafficking pathway, involving the fusion of multivesicular endosomes or bodies (MVEs/MVBs) with the plasma membrane. This event leads to the extracellular release of the intraluminal vesicles (ILVs) contained in the MVEs generating a subtype of small EVs (30–100 nm) usually referred to as “exosomes” (Figure 1). The microvesicle/exosome nomenclature has been lately considered controversial, since there are not specific markers to clearly distinguish each EV biogenesis pathway. Therefore, following the recommendations stated by the positional paper of the International Society for Extracellular Vesicles (ISEV) in 2018, EVs should rather be named according for instance to their size: small EVs (<100 nm or <200 nm) and medium/large EVs (>200 nm) [25].

Irrespective of their origin, all EVs are circular membrane vesicles that contain cytosolic components and express surface adhesion molecules characteristic of their cells of origin. Specific differences in EV size and/or in surface molecules may influence their recognition and capture by target cells. EVs can be taken up nonspecifically by two endocytic means, phagocytosis and macropinocytosis (Figure 1). Alternatively, EVs can be captured by other canonical forms of endocytosis through specific receptor-ligand interaction (clathrin or caveolin-mediated) [26]. EV may also deliver their cargo by fusion with the plasma membrane [26]. Once the vesicle is internalized, its content can be degraded or released into the cytoplasm and transported into the nucleus or cellular membranes, leading to functional consequences. Additionally, interaction of EV membrane proteins with receptors of the recipient cell plasma membrane may lead to stimulation of intracellular signaling cascades (Figure 1). All these specific and nonspecific mechanisms of EV capture and interaction open a wide spectrum of possibilities for EV-mediated intercellular communication, such as the transfer of enzymes, receptors, transcription factors, oncogene products, infectious particles, mRNAs and miRNAs (which can induce epigenetic modifications in the recipient cells), and the stimulation of the target cells through interaction with cell surface-expressed receptors [27].

The fact that EVs are molecularly reflective of their tissue of origin is particularly important in the context of cancer, since cancer cells are known to produce greater numbers of EVs compared to healthy cells and they contain molecules specific of their neoplastic origin. This is relevant in order to use EVs as cancer biomarkers but also to design potential therapeutics to target tumor-derived EVs in order to avoid cancer dissemination or to convert EVs into vehicles for the delivery of therapeutic molecules.

## 3. Extracellular Vesicles Released by Glioblastoma Modulate the Tumor Microenvironment

The GBM microenvironment consists of diverse cellular populations, including tumor cells, endothelial cells and pericytes of blood vessels; innate immune cells (microglia, monocytes, macrophages, neutrophils and mast cells), T cells, neurons, astrocytes, oligodendrocytes and GSCs; and extracellular matrix components [8].

It is well known that GBMs interact with surrounding non-cancer cells to maintain a microenvironment that favors tumor proliferation, invasion of the brain, angiogenesis and immunosuppression. Multiple modes of communication are involved in this phenomenon, such as soluble factors, gap-junctional communication, connecting nanotubes and EVs (reviewed in [8]). EVs constitute an outstanding communication system in the sense that they can deliver not only soluble proteins, but a wide variety of coding and non-coding RNAs that can alter gene expression in recipient cells [28,29,30]. EVs have been involved in the mechanisms of tumor progression and invasion in different types of cancers [16]. Their relevance in GBMs is being object of study in the last years, as they might be partially responsible for the high degree of recurrence of this cancer type, as well as for their interest as GBM biomarker.

We will describe below relevant findings showing effects of EVs released by GBMs on different component of the tumor microenvironment, specifically on immune cells, vasculature, astrocytes and GSCs.

### 3.1. Effects on Monocytes, Macrophages and Microglia

Infiltrating monocytes, macrophages and microglia are the most prevalent cell types within the GBM microenvironment [31]. They used to be termed together as tumor-associated macrophages (TAMs), although they represent different cell populations [32]. Microglia are the resident innate immune cells in the brain and are important in the defense against pathogens and noxious stimuli [33]. Disruption of the blood brain barrier induced by the GBM leads to infiltration of monocytes that can subsequently differentiate to macrophages [32]. Macrophages can acquire different phenotypes according to cytokines and signaling molecules of the microenvironment [34,35]. Macrophages with the classical M1 phenotype are capable of phagocytosis, cytotoxicity, antigen presentation and the promotion of inflammation. In contrast, in response to GBM-derived soluble factors, macrophages can acquire a so-called M2 phenotype in which they produce chemokines, cytokines, growth factors, angiogenic factors, extracellular matrix-modifying enzymes and immunosuppressive molecules, which make the environment favorable to the tumor progression [36]. Evidence from the last few years show that EVs released by GBM cells intervene in the induction of such tumor-supportive TAMs’ phenotype modulation. In an article published in 2015, de Vrij et al. [37] reported that EVs derived from GBMs (U87MG GBM cell line and GBM primary cultures) were able to modify the phenotype of TAMs in vitro, changing it to a M2-like anti-inflammatory phenotype that resembles the tumor-supportive phenotype observed in patients. Phenotypic changes included modified expression of a wide variety of cell surface molecules (e.g., increased CD14 and reduced HLA-DR), and increased secretion of cytokines such as interleukin-6 (IL-6) and vascular endothelial growth factor (VEGF). In addition, GBM EVs induced and increased phagocytic activity of macrophages, which may enhance degradation of extracellular matrix and facilitate the migration of tumor cells.

**Figure 2:**
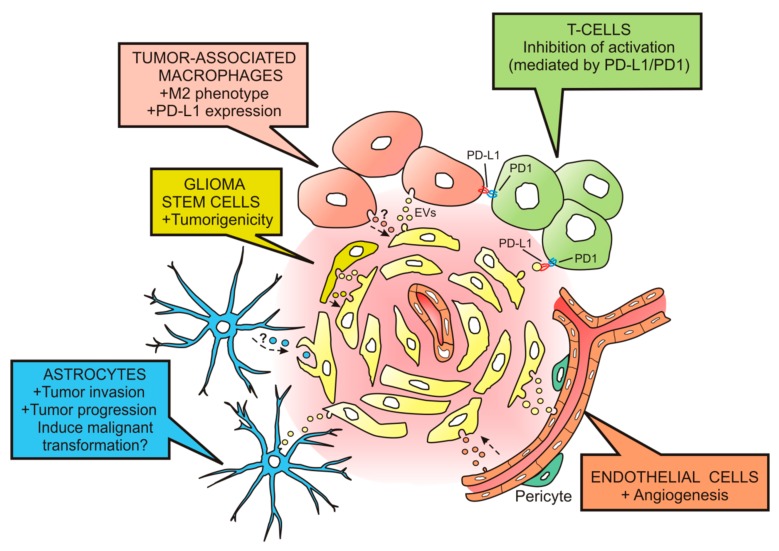
Effects of glioblastoma-derived extracellular vesicles on cells from the tumor microenvironment. Extracellular vesicles (EVs) released by glioblastoma (GBM) cells (in pale yellow) can be taken up by tumor-associated macrophages (in flesh color) to induce the M2 phenotype and the expression of PD-L1. By interacting with the ligand PD1 expressed on activated T cells (in green), they inhibit the T cell-associated immune response. Additionally, EVs can target T cells and suppress their activation through PD-L1/PD1 interaction. Endothelial cells (in orange) are also targets for GBM-derived EVs; they activate angiogenic mechanisms. The uptake of GBM EVs by astrocytes (in blue) leads to astrocytic responses favoring tumor invasion and progression. The malignant transformation of astrocytes might also be produced by EVs delivering oncogenic cargo. GBM-derived EVs can also deliver their cargo in glioma stem cells (in dark yellow) producing an increase in their tumorigenicity. Tumor microenvironment components might also release EVs to induce effects on the GBM cell population (see dashed arrows).

One year later, van der Vos et al. [38] demonstrated efficient uptake of EVs released by GBM cells (GL261 GBM cell line and primary human GBM cells) by TAMs, both in vitro and in vivo. In addition, they showed that miRNAs contained in these vesicles were transferred to the recipient TAMs, resulting in downregulation of target mRNAs. The same group recently demonstrated functional delivery of miRNA-21 (mi-R21) contained in EVs from the GBM cell line GL261 to surrounding microglia, subsequently leading to downregulation of specific miR-21 mRNA targets [39]. One of these targets is the Btg2 gene, which exerts antiproliferative effects. Consequently, microglial cells might increase their proliferation and their influence on shaping the tumor microenvironment. Related experiments performed by Gabrusiewicz and colleagues [40] showed that exosomes, isolated among the EVs released by the GBM cell line U87 and by GSCs, mainly target monocytes to induce reorganization of the actin cytoskeleton and the immune suppressive M2 phenotype, with the secretion of cytokines such as monocyte-chemotactic protein 3 (MCP-3) and chemokine (C-X-C motif) ligand 1 (CXCL1), and the expression of the programmed death-ligand 1 (PD-L1). The expression of PD-L1 by monocytes may be responsible for cancer-induced immune evasion, since PD-L1 can bind to programmed cell death protein 1 (PD1) of activated T cells to inhibit T cell function [41] (Figure 2).

In this regard, researchers have tried to modify the tumor-supportive phenotype of TAMs by means of exogenous EVs. This has been performed with EVs released from microglia in inflammatory conditions. The injection of these vesicles in glioma-bearing mice reduced the anti-inflammatory phenotype of TAMs and significantly reduced tumor size and tumor induced neurotoxicity [42]. These results are important in terms of achieving valuable approaches to transfer signals to TAMs to restore the homeostatic microglial phenotype.

### 3.2. Effects on T Cells

T cells can also be the targets of EVs released by primary human GBM cells, and their activation can be blocked as a result of such interaction [42]. This interaction is probably achieved through PD-L1/PD1, since PD-L1 is found in GBM EVs and the EV-induced inhibition of T cell activation is partially reversed by PD1 blockade [43]. These results indicate that PD-L1 expression on EVs may be an additional mechanism for GBM to suppress antitumor immunity (Figure 2).

### 3.3. Effects on Endothelial Cells

GBMs are among the most vascularized tumors, characterized by microvascular proliferation and endothelial hyperplasia. Indeed, these features are used as histological markers to define high-grade gliomas [44]. These regions of angiogenesis are critical for tumor progression and are key locations for GSCs [45,46]. Glioma cells release soluble factors that contribute to the shape of tumor vasculature, such as VEGF-A [47], stromal-derived factor-1 [48] and hepatoma-derived growth factor [49].

The laboratory of Xandra O. Breakfield was the first one to demonstrate that human patient GBMs produce EVs containing mRNA, miRNA and angiogenic proteins that can be taken up and translated by cultured endothelial cells to stimulate angiogenesis [28] (Figure 2). This work pioneered with the assumption of EVs acting as vehicles to deliver genetic information and proteins to recipient cells in the GBM microenvironment. They identified several mRNAs and miRNAs in GBM-derived EVs, among which the epidermal growth factor receptor (EGFR) mRNA is of particular interest since the expression of the EGFR mutant/variant III (EGFRvIII) is characteristic of a clinical subtype of GBM [50]. This raised expectations for the use of EVs as diagnostic markers for GBM.

The ability of GBM-derived EVs to modify the phenotype of endothelial cells has been reported thereafter by other groups. For instance, Li et al. described changes in gene expression in cultured vascular endothelial cells exposed to EVs released from U251 glioma cells [29]. EVs produced by primary GBM cells grown at hypoxic conditions are potent inducers of angiogenesis ex vivo and in vitro, and through mechanisms derived of EV-induced phenotypic modulation of endothelial cells [51]. Moreover, endothelial cells programmed by GBM-derived hypoxic EVs secrete cytokines and growth factors that stimulate pericyte migration and PI3K/AKT signaling [51]. These findings indicate that EVs are mediators of hypoxia-dependent intercellular communication between GBM and vascular cells. 

Molecules delivered by EVs that have been involved in the promotion of angiogenesis are: VEGF-A [47]; miR-21, which in turn upregulates VEGF expression [52]; transforming growth factor β (TGFβ); and proteolytic enzymes [53] or long non-coding RNA CCAT2 [54], which induces upregulation of VEGF-A and TGFβ in cultured endothelial cells. EVs from patient-derived GBM cells can exert additional effects on vasculature, such as an increase in vascular permeability associated to the expression of the pro-permeability factor Semaphorin3A in EV surface [55]. 

### 3.4. Effects on Astrocytes

Among all the cell types surrounding GBMs, astrocytes are the phenotypically most similar to the bulk of this tumor. Although it is well known that peritumoral astrocytes may contribute to glioma progression, not much has been reported on the effects induced by GBM cells on the modulation of astrocyte function to contribute to tumor support [56,57]. Chen et al. showed that brain metastatic cells can assemble to astrocytes through gap junctions composed of connexin 43. Once engaged with the astrocytic gap-junctional network, cancer cells transfer the second messenger cGAMP to astrocytes. This messenger activates the astrocytic production of inflammatory cytokines such as interferon-α and tumor necrosis factor, which in turn induce the activation of STAT-1 and NF-κβ pathways in the brain’s metastatic cells, thereby supporting tumor cell invasion and resistance to chemotherapy [58]. More recently, two articles have reported tumor-supportive effects of astrocytes driven by EVs released by GBMs [59,60]. Oushy et al. demonstrated that astrocytes cultured with EVs from patient-derived GBMs display profound modifications of the secretome that results in positive effects on tumor growth [59]. These include increased production of growth factors (EGF, VEGF, colony stimulating factors (CSFs), fibroblast growth factors and hepatocyte growth factor), chemokines (CXCL1, 9, 10 and 11; chemokine C-C motif ligand 3, 5, 7 and 20), matrix metalloproteinase-9; and some cytokines that could induce immune suppression, such as CSF2 and 3, and ILs 4, 10 and 13. 

In a later study, Hallal et al. [60] reported increased podosome formation and extracellular matrix degradation in astrocytes cultured with GBM-derived EVs. In addition, they showed that the proteome of astrocytes exposed to GBM-derived EVs presented significant modifications in signaling pathways involved in tumorigenesis, such as decreased TP53 and increased MYC. TP53 is a tumor suppressor gene that encodes for the sequence-specific DNA-binding protein p53. This protein, p53, is important in the cell response to genotoxic stress, since it induces apoptosis or cell cycle arrest, thereby blocking the transmission of DNA mutations to progeny cells [61]. TP53 mutations leading to p53 loss are frequent in GBM [62]. Results from previous studies suggest that the p53 status of GBM cells may affect the microenvironment to promote tumor progression. For instance, astrocytes grown with GBM cell conditioned media show decreased p53 levels which correlates with a modulation in extracellular matrix composition to favor tumor malignancy [63]. MYC is a proto-oncogene that codes for Myc proteins, which act as transcription factors involved in cell cycle regulation, apoptosis, proliferation and differentiation, with important roles in acquisition and maintenance of stem cell properties, including in GSCs [64,65]. Interestingly, transduction of p53(–/–) astrocytes with Myc induces tumorigenic abilities [66], an effect also observed in astrocytes transformed with a combined transduction of Myc, a dominant negative form of p53, Oct-4 and H-ras [67]. All these findings reported in in vitro experiments suggest that GBM-derived EVs may stimulate astrocytes to promote the invasion and expansion of GBMs also in vivo (Figure 2).

The relevance of these findings is not only that GBM EVs could modify neighboring astrocytes to induce tumor-supportive functions, but, importantly enough, GBM EVs may drive astrocytes to become tumorigenic themselves. GBM EVs’ contents include oncogenic proteins and regulators, such as EGFRvIII (mRNA and protein), miR-10b or miR-21 [23,38,68], which could induce the malignant transformation of astrocytes. Indeed, in the above mentioned study by Oushy and colleagues [59], GBM EVs drove significant molecular changes in astrocytes that resemble cancer signaling pathways, and reduced astrocyte dependence on anchorage to a solid matrix, an indicator of cellular transformation. In line with this, it is important to highlight that astrocyte-like stem cells of the neurogenic niche of the subventricular zone have been considered a putative origin of human GBMs [69]. It is therefore feasible that astrocyte-like stem cells of the subventricular zone may acquire GSC features as a result of receiving instructive signals from EVs of variable origin. It is worth mentioning the recent study by Wang and colleagues [70] in which they demonstrate that GBM-derived EVs induce the transformation of human embryonic neural stem cells into tumor-promoting cells. 

### 3.5. Effects on Glioma Stem Cells

GBMs harbor stromal cells resembling mesenchymal stem cells [71] that have been involved in GSC tumorigenicity by mechanisms depending on the secretion of IL-6 [72]. A subsequent study revealed that GBM mesenchymal stem cells from surgical specimens also release EVs that increase the proliferation and clonogenicity of GSCs [73] by transfer of miR-1587, which in turn induces a decrease in the expression of the tumor suppressor NCOR1 (nuclear hormone receptor corepressor-1). Therefore, it is important to take into account that GSCs are targets for EVs released within the GBM microenvironment, and, at the same time, GSCs release EVs which can induce protumorigenic effects on GBM cells (Figure 2). For instance, Palumbo et al. [74] have shown that EVs released by GSCs exert a stimulatory effect on cell growth and migration of the adherent GBM cell line U87MG. GSC-derived EVs decorated with the cell adhesion molecule L1CAM are also able to induce motility, proliferation and invasiveness of different types of GBM cell lines [75]. 

Interestingly, these GSC-GBM exchange of EVs might be involved in the well known intratumoral heterogeneity characteristic of GBMs. Ricklefs et al. demonstrated that EVs released by GSCs retain tumor subtype characteristics and that their transfer between GSC subtypes leads to protumorigenic changes in the target GSC [76].

### 3.6. Effects on Other Cell Populations of the Tumor Microenvironment

To date, no evidence has been published on the effects mediated by EVs released by GBM on neighboring oligodendrocytes or neurons, although such effects can not be discarded.

### 3.7. Technical Considerations

Isolation of EVs from conditioned media or biological fluids can be achieved to various degrees through different techniques such as ultracentrifugation, ultrafiltration, chromatography, polymer-based precipitation, affinity capture on antibody-coupled magnetic beads and microfluidic devices [77]. They all have advantages and disadvantages, and still, an ideal and universal method for EV isolation is lacking. The studies mentioned in this section have used ultracentrifugation-based methods such as differential ultracentrifugation [28,29,40,43,51,52,59,60,73] or density gradient ultracentrifugation [37,38,39]. These are the most used technical procedures (at least in 2018 [77]), and have been validated by the ISEV2018 position paper [25]. 

To characterize isolated EVs (size distribution and concentration), most reports showing effects of GBM EVs on tumor microenvironment used nanoparticle tracking analysis, with the exception of de Vrij and colleagues [37] that performed the EV quantification by tunable resistive pulse sensing. Some of these studies also performed transmission electron microscopy to further characterize the EV structure, morphology and size [60,73]. 

## 4. Extracellular Vesicles Released by Tumor Microenvironment Cells Might Also Modulate Glioblastoma Cells

Although most work has been carried out on the effects of GBM-derived EVs on the tumor microenvironment, the reverse phenomenon is slowly gaining importance. That is, tumor environ cells might in turn release EVs that could be taken up by GBM cells to mediate effects such as proliferation, migration or immunosuppression. Some recent papers support this hypothesis. The close proximity in the perivascular niche of endothelial cells with GSCs suggests that endothelial cell-derived EVs may target GSCs. It has been previously reported that endothelial cells can transfer miRNA to breast cancer cells via exosomes with induced modifications in their proliferative and invasive capacities [78]. Based on this background, Li et al. [79] analyzed the effects of EVs secreted by human endothelial cells on GSC proliferation and self-renewal. They demonstrated that treatment with endothelial cell EVs promotes GSC tumorigenicity through mechanisms derived of the transfer of CD9, a transmembrane protein involved in the regulation of cell fusion, adhesion and motility that is relevant for the maintenance of GSCs [80]. 

EVs released by reactive astrocytes could also enter GBM cells. In favor of this idea Zhang et al. [81] reported astrocyte-derived EV transfer of microRNAs targeting the tumor suppressor protein PTEN to brain metastatic breast cancer and melanoma cells. The epigenetic PTEN loss induced by this intercellular transfer to brain metastatic cells led to enhanced tumor proliferation and reduced apoptosis. TAMs might also release EVs and target GBM cells. For instance, in gastric cancer cells, there is an EV-mediated transfer of miR-21 from TAMs to tumor cells, which also downregulates PTEN and suppresses apoptosis [82].

Future work will reveal whether EVs released by GBM microenvironment cells, such as endothelial cells, TAMs or astrocytes, have an impact on GBM growth, progression and resistance to therapy, and will evaluate the relevance of these positive feedback loops of cellular communication.

## 5. New Perspectives

The facts that EVs can be released into biological fluids and that their contents are reflective of the cellular origin have enabled their use as cancer biomarkers. Thus, the analysis of the cargo of EVs purified from serum or cerebrospinal fluid is emerging as a powerful tool for GBM diagnosis, subtype identification and assessment of tumor resistance to therapies [83,84]. However, EVs’ therapeutic importance in GBMs extends beyond their use as biomarkers, and currently they are beginning to be considered for antitumoral therapy. The therapeutic application takes advantage of the EV ability to cross the blood brain barrier and consists of encapsulating therapeutic cargo in EVs to deliver messages aimed at suppressing oncogenicity of GSCs or inhibiting tumor-supportive actions of microenvironment cells [85]. In addition, knowing the important roles played by EVs in the maintenance of a tumor-supportive environment, in the tumorigenicity of GSCs or even in the induction of malignant transformation, it is logically sound to think that future strategies will be focused in trying to suppress EV biogenesis or EV release by GBM cells.

## 6. Conclusions

EVs constitute one of the multiple ways through which GBM cells communicate with the tumor microenvironment for their own benefit. GBM-derived EVs can be taken up by different components of the tumor environment, such as TAMs, endothelial cells, astrocytes and GSCs. EV cargo delivery in these cells leads to effects involved in tumor growth, tumor invasion, neo-angiogenesis, oncogenic transformation and modulation of the immune response. In turn, cells from the tumor environs also release EVs that may target GBM cells to increase tumorigenicity. 

Future therapeutic strategies for GBM could aim at interrupting this EV-mediated cooperative crosstalk established between GBM cells and their stromal counterparts.
